# A Relevant Wound-Like *in vitro* Media to Study Bacterial Cooperation and Biofilm in Chronic Wounds

**DOI:** 10.3389/fmicb.2022.705479

**Published:** 2022-04-06

**Authors:** Cassandra Pouget, Catherine Dunyach-Remy, Thierry Bernardi, Christian Provot, Jason Tasse, Albert Sotto, Jean-Philippe Lavigne

**Affiliations:** ^1^Virulence Bactérienne et Infections Chroniques, INSERM U1047, Université de Montpellier, Nîmes, France; ^2^Biofilm Pharma SAS, Saint-Beauzire, France; ^3^Virulence Bactérienne et Infections Chroniques, INSERM U1047, Université de Montpellier, Department of Microbiology and Hospital Hygiene, CHU Nîmes, Nîmes, France; ^4^Virulence Bactérienne et Infections Chroniques, INSERM U1047, Université de Montpellier, Department of Infectious and Tropical Diseases, CHU Nîmes, Nîmes, France

**Keywords:** bacterial cooperation, biofilm, chronic wound, *in vitro* medium, nematode killing assay, *Pseudomonas aeruginosa*, *Staphylococcus aureus*, virulence

## Abstract

Biofilm on the skin surface of chronic wounds is an important factor in the pathology, inhibiting wound healing. The polymicrobial nature of these infected wounds and bacterial interactions inside this pathogenic biofilm are the keys for understanding chronic infection. The aim of our work was to develop an innovative *in vitro* medium that closely mimics the chronic wound emphasizing the microbiological, cellular, and inflammatory environment of chronic wounds but also focusing on the pH found at the wound level. This new medium, called chronic wound medium (CWM), will thus facilitate the study of pathogenic biofilm organization. Clinical *Staphylococcus aureus* and *Pseudomonas aeruginosa* strains coisolated from diabetic foot infection were collected and cultivated in this new medium for 24 h in monoculture and coculture. Bacterial growth (growth curves), presence of small colony variant (SCV), biofilm formation (BioFilm Ring Test^®^ assay, biofilm biomass quantification), and virulence (survival curve in a *Caenorhabditis elegans* model) were evaluated. After 24 h in the *in vitro* conditions, we observed that *P. aeruginosa* growth was not affected, compared with a control bacterial medium, whereas for *S. aureus*, the stationary phase was reduced by two logs. Interestingly, *S. aureus* growth increased when cocultured with *P. aeruginosa* in CWM. In coculture with *P. aeruginosa*, SCV forms of *S. aureus* were detected. Biofilm studies showed that bacteria, alone and in combination, formed biofilm faster (as soon as 3 h) than the bacteria exposed in a control medium (as soon as 5 h). The virulence of all strains decreased in the nematode model when cultivated in our new *in vitro* medium. Taken together, our data confirmed the impact of the chronic wound environment on biofilm formation and bacteria virulence. They indicated that *P. aeruginosa* and *S. aureus* cooperated in coinfected wounds. Therefore, this *in vitro* model provides a new tool for bacterial cooperation investigation and polymicrobial biofilm formation.

## Introduction

Chronic wounds are a major public health challenge worldwide. Skin diseases represent an important human issue as well as an economic burden (Järbrink et al., 2017). The incidence of chronic wounds is likely to be underestimated. These pathologies are becoming increasingly frequent because of an aging population and earlier development of chronic illnesses such as diabetes mellitus, among others (Sen et al., 2009). Recent estimations suggest that more than 7 million people are currently affected in the United States and 4 million people in Europe, and this figure is expected to rise by 2% in the next decade (Sen, 2019). Chronic wounds are defined by four types of pathologies: diabetic foot ulcers (DFUs), vascular ulcers (containing venous and arterial ulcers), and pressure ulcers (Kirsner, 2016). Infections are a common complication of these ulcers delaying healing. They are associated with substantial morbidities, requiring frequent health care provider visits, daily wound care, antimicrobial therapy, and surgical procedures with associated high health care costs (Schaper et al., 2020). The difficulty in treating those non-healing wounds is partially caused by the polymicrobial nature of the skin bed (Bowler et al., 2001), in which microorganisms are organized in pathogenic biofilms (Dowd et al., 2008). In order to better decipher biofilms and to find new antibiofilm approaches, social traits of bacteria and their interactions inside the matrix of extracellular polymeric substances need to be explored. The most frequent, and studied, interaction described in chronic wounds is that between *Staphylococcus aureus* and *Pseudomonas aeruginosa*, because of the high prevalence of both species in these wounds (Serra et al., 2015). Some studies, especially in a cystic fibrosis context, have shown that *P. aeruginosa* quickly killed *S. aureus* when grown together *in vitro* (Palmer et al., 2007). This killing is due to exoproducts of *P. aeruginosa*, such as 4-hydroxy-2-heptylquinoline-N-oxide (Hoffman et al., 2006), the Pel and Psl products (Qin et al., 2009), and pyocyanin (Dietrich et al., 2006). However, more recent data suggest that the interaction between the species could be symbiotic, with, for example, the secretion of *P. aeruginosa* alginate having a protective effect on *S. aureus* (Price et al., 2020; Schurr, 2020). So far, very few data are available concerning chronic wounds. The main hypothesis is that both microorganisms are present, but occupy distinct regions of the wound without interacting (Fazli et al., 2009). Recently, Alves et al. (2018) observed interactions between the two species influencing both colonization and pathogenicity in this clinical situation. The only *in vitro* model developed for the study of multispecies biofilms at the wound level was the Lubbock model (Sun et al., 2008). DeLeon et al. (2014) used it to study interactions between *P. aeruginosa* and *S. aureus* inside the polymicrobial biofilm. However, in all these studies, the experiments were performed under conditions that did not consider the “clinical” environment of the chronic wounds. Indeed, to our knowledge, no culture media has been used to study microorganisms closer to *in vivo* conditions considering wound parameters such as pH, temperature, cellular and inflammatory environment, the presence of serum, or the wound irrigation. The aims of our work were to develop a new affordable, convenient, and reliable *in vitro* medium to study the bacterial interactions in the wound environment encountered in chronic wounds and to investigate their role inside the biofilm.

## Materials and Methods

### Bacterial Strains and Culture Conditions

All bacterial strains used in this study are listed in Table 1.

**TABLE 1:** Strains used in this study.

Strain	Characteristics	References
PAO1	*P. aeruginosa* reference strain	Watters et al., 2015
Newman	*S. aureus* reference strain	Wang et al., 2020
SAC1	Clinical strains of *S. aureus* coisolated with PAC1 from a diabetic foot ulcer (grade 3)	In this study
PAC1	Clinical strains of *P. aeruginosa* coisolated with SAC1 from a diabetic foot ulcer (grade 3)	In this study

The *in vitro* chronic wound medium (CWM) is composed of the following: 79.5% of Bolton broth, 20% of heat-inactivated human serum, and 0.5% of hemolyzed human blood (Table 2). The Bolton broth (Sigma-Aldrich) was prepared following manufacturer’s recommendations. Human frozen serum and human blood were purchased from Etablissement Français du Sang, which guarantees that these samples have been obtained from healthy donors. Serum serotyping has been evaluated. The first step of the serum preparation consisted of a slow overnight thawing at 4°C followed by aliquoting. Decomplementation of the serum was then performed at 56°C for 30 min. After cooling, serum was either filtered on 0.22 μm for immediate utilization or frozen for conservation. The first step of the blood preparation consisted of aliquoting before blood hemolysis. For this step, blood samples were frozen at −20°C and then thawed at room temperature for 2 h. We performed cycle of freezing/thawing three times before decantation and pellet removal. Hemolyzed blood was then either filtered on 0.22 μm for immediate utilization or frozen for conservation. Sterile Bolton broth was then mixed with filtered human hemolyzed blood and human decomplemented serum in the concentration described above. This medium, adapted from Sun et al. (2008), was complemented by adding 1.10^6^/mL of human keratinocyte debris (HaCaT cells). HaCaT cells were purchased from the ATCC. The cells were grown in Dulbecco modified eagle medium, high glucose, GlutaMAX™ Supplement (Thermo Fisher Scientific, Illkirch, France) with 10% decomplemented serum (BioWhittaker, Walkersville, MD). HaCaT cells were lysed to obtain human keratinocyte debris by physical disruption. Aliquoted cells were immersed and frozen in a liquid nitrogen bath for 2 min. The aliquots were then thawed at room temperature for 1 h. This freezing/thawing cycle was carried out three times. Cell viability and size and number of cell debris obtained were controlled by FACS analysis. Finally, the pH was adjusted to 8.0 by buffering with 10 mM HEPES/NaOH. By adding serum and blood to the Bolton broth, the CWM mimics the three major constituents of the chronic wound bed: red blood cells, serum, and damaged tissues.

**TABLE 2:** Composition of the chronic wound medium (CWM).

CWM composition
79.5% Bolton broth vol/vol
20% Heat-inactivated human serum vol/vol
0.5% Hemolyzed human blood vol/vol
1 × 10^6^/mL debris of human keratinocytes (HaCaT)
NaOH 1 M for a fixed pH 8
1% 1 M HEPES

Bacteria were grown in bacterial culture tubes or in Erlenmeyer culture flasks under shaking at 200 revolution/min, 37°C in brain–heart infusion (BHI; Sigma-Aldrich) broth, or in CWM.

### Growth Curves

Samples of the bacterial growing culture were collected at different time points, diluted in 1 × phosphate-buffered saline (PBS; Thermo Fisher Scientific, Gibco), and plated on non-selective agar [Luria–Bertani (LB) agar; Thermo Fisher Scientific] in monoculture or in selective agar when bacteria were grown in coculture (mannitol salt agar for *S. aureus*, Oxoid; and cetrimide agar for *P. aeruginosa*, Oxoid) to evaluate the number of colony-forming units (CFU)/mL. Cocultures were initiated 1:1 starting with 4.10^4^ CFU/mL. Cultures were grown under the same conditions, with the same microbiological culture flask under static and aerobic environment at 37°C for 24 h. Each experiment was performed in triplicate.

### Phenotypic Characteristics of the Strains

To investigate the impact of the coculture on the strains physiology, the bacteria were plated before and after coculture in BHI or CWM. Twenty microliters of the suspension were inoculated onto tryptic soy blood agar (Sigma–Aldrich), without antibiotics, and incubated 48–72 h at 37°C. Colonies were described after 48 and 72 h according to presence/absence of β-hemolysis (for *S. aureus*) (Zhang et al., 2016), colony morphology (Proctor et al., 2006), and characteristic pigment production (for *P. aeruginosa*) (Howarth and Dedman, 1964). Both *S. aureus* and *P. aeruginosa* with a distinctive morphology [compatible with small colony variants (SCVs); Proctor et al., 2006] were subcultured onto tryptic soy blood agar plates 48–72 h at 37°C to evaluate the reversibility of the colony morphology. The percentages of SCV, hemolysis, and pigmentation were calculated by counting the number of bacterial colonies exhibiting the phenotypic characteristics described and comparing it with the total of colonies present on the dish plate. Each experiment was performed in triplicate.

### Kinetics of Early Biofilm Formation

The early biofilm formation was assessed using BioFilm Ring Test^®^ (BioFilm Control, Saint Beauzire, France), as previously described (Chavant et al., 2007) and according to the manufacturer’s recommendations. This assay allows the observation of the dynamic immobilization of superparamagnetic microbeads embedded in biofilms. *P. aeruginosa* and *S. aureus* strains were subcultured on BHI agar (control) and CWM agar at 37°C for 24 h. Six colonies were inoculated into BHI broth as the control condition or in CWM and homogenized. The bacterial suspension was standardized to an optical density at 600 nm of 1.00 ± 0.05 and diluted 1:250 in BHI broth or CWM (without human keratinocyte debris) to obtain a final concentration of 4.10^6^ CFU/mL using a defined calibration curve between OD and CFU/mL. This bacterial suspension was complemented with 1% (vol/vol) magnetic beads (TON004). Two hundred microliters was then added, in triplicate, into a 96-well microplate (Falcon 96 Flat Bottom Transparent, Corning, United States) for each time point (1, 3, and 5 h). The plates were incubated without shaking at 37°C. After incubation, 100 μL of liquid contrast (LIC001) solution was added on the top of each well. The microplate was placed on a magnetic block for 5 min and scanned using the Biofilm Control plate reader and the BFCE3 software provided by the company. Each experiment was performed twice in triplicate. A negative control was systematically included in each experiment corresponding to the medium and beads without bacterial suspension.

Spots were quantified through specialized image algorithms that measure bead aggregation. Biofilm index (BFI) values were calculated for each well, ranging from 0 (no aggregation, i.e., biofilm formation) to 20 (total aggregation, i.e., absence of biofilm formation). A BFI < 2 represents the cutoff for fixed biofilm. As cellular debris can interact with the paraelectromagnetic beads used in the assays and cause false negatives, the CWM used for BRT experiments was decomplemented of human keratinocyte debris. The preculture of the strains was therefore carried out in a complete medium (with cellular debris). The behavior of the strains reflects an adaptation to the medium used in preculture and not to the medium used for the BRT due to the short time of the incubation.

### Quantification of Biofilm Living Biomass

The mature biofilms were evaluated using living bacteria encountered inside biofilm quantification (Freitas et al., 2014). The optical density at 600 nm of overnight bacterial suspensions, in BHI broth or CWM, was adjusted to 1.00 ± 0.05, before a 1:100 dilution in BHI broth or CWM. Two hundred microliters of each suspension was transferred to a microplate (Falcon 96 Flat Bottom Transparent, Corning, United States) in triplicate and incubated at 37°C for 24 h without shaking. Negative control wells contained BHI broth or CWM alone. After incubation, the microplates were washed three times with 200 μL of 1 × PBS. Two hundred microliters of 1 × PBS was finally added into the well before biofilm disruption. The disruption was performed by sonication for 10 min at 40 kHz. Each well was then 10-fold serially diluted, and the last dilution was plated on non-selective agar (LB agar) or in selective agar when bacteria were grown in coculture (mannitol salt agar for *S. aureus* and cetrimide agar for *P. aeruginosa*). The agar plate was then incubated overnight at 37°C, and CFUs were counted. The experiment was performed twice for each sample. Experiments were performed with either bacteria alone or in coculture.

### Nematode Killing Assay

The nematode infection assay was performed as described previously (Lavigne et al., 2008) using the Fer-15 mutant line, a temperature-sensitive fertility defect. Overnight cultures of *S. aureus* and *P. aeruginosa* strains in BHI broth or CWM were incubated at 37°C under shaking. One hundred microliters of the bacterial suspension was spotted on nematode growth medium agar. A control with the *Escherichia coli* OP50 strain was used for nematodes. This bacterium is the standard feeding strain for Fer-15 as it shows no pathogenic virulence factors. Approximately 30 L4 stage nematodes were then seeded on each plate and incubated at 25°C. Every day, an independent reader scored the number of alive nematodes under a stereomicroscope (Leica, France). The lethal time 50% (LT50), which corresponds to time (in days) required to kill 50% of the worms, was calculated.

### Statistical Analysis

*T*-test was used to compare the *in vitro* bacterial growth of the different strains as well as the comparison of the early kinetics of biofilm formation and biofilm biomass. To compare overall survival curves in nematode killing assays, a Cox regression was used. For pairwise comparison of two survival curves in nematode killing assays, we used a log-rank test. Statistical analyses were performed using GraphPad Prism version 7 or R version 3.5.2. Tests used for the *p*-value determination are mentioned in each figure legend.

## Results

### Modification of *Staphylococcus aureus* Growth in the *in vitro* Chronic Wound Medium

**FIGURE 1:**
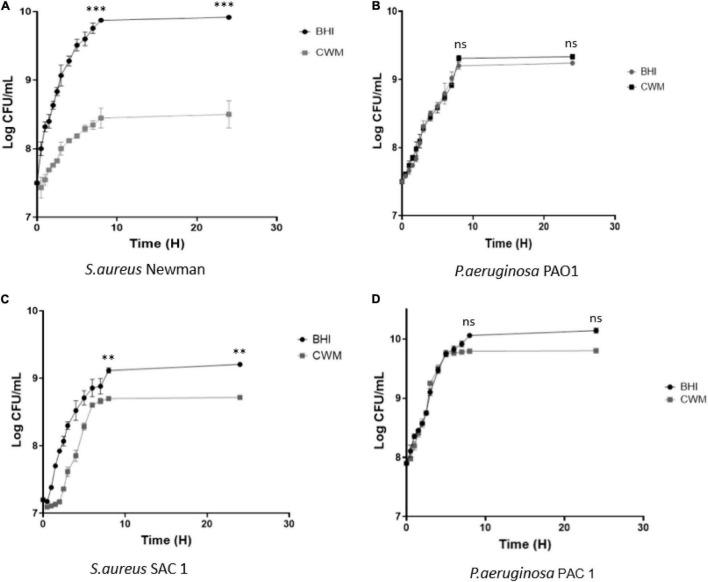
Growth curves of reference **(A,B)** and clinical **(C,D)** strains of *S. aureus* and *P. aeruginosa* in CWM and BHI media. Cultures were sampled at the indicated time points, and the numbers of bacteria were estimated by CFU enumeration of *P. aeruginosa* and *S. aureus* on non-selective medium. Experiments were performed in three biological replicates; data points represent the average of these replicates, and error bars represent the standard deviation. Comparisons were performed with *t*-test. Significance was set to a *p* < 5%. ns, not significant; ***p < 0.001, **p < 0.01.

CWM is a medium developed to mimic the *in vivo* conditions encountered in chronic wounds. To evaluate its effect on bacteria, we first compared the bacterial growth of two of the main microorganisms isolated from chronic wounds in this new medium and in a usual microbiological medium (BHI). Concerning *S. aureus*, we noted that the reference strain Newman presented a significant decrease in the growth curves at the early exponential phase in the CWM (Figure 1A) compared with BHI (*p* < 0.001) and a difference of one log once the stationary phase reached (*p* < 0.001). The same profile was observed for the clinical strain SAC1, with a clear delay of the early exponential phase and a significant difference of 0.5 log at stationary phase of bacterial growth in the CWM versus BHI (*p* < 0.01) (Figure 1C).

For *P. aeruginosa*, no difference was observed in growth curves for the reference strain PAO1 in BHI or CWM medium (Figure 1B). The clinical strain PAC1 had a reduced growth rate of the last part of the exponential phase (after 8 h) and a slight difference of 0.2 log once the stationary phase reached [*p* = 0.0874, not significant (NS)] (Figure 1D).

The phenotypic modifications of the bacteria are presented in Supplementary Table 1. Globally, cultures in CWM showed significantly more SCVs and a loss of β-hemolysis by *S. aureus* and of pigmentation by *P. aeruginosa* (*p* < 0.01).

In order to test the possibility of a prolonged culture of bacteria to mimic the chronicity of a wound, we also cultivated the bacteria for 6 weeks with weekly renewal of the medium. Supplementary Table 2 shows the log of the maximal bacterial density obtained after 6 weeks and compared with those after an adaptation and culture in the same medium for 24 h. After 6 weeks of culture in the CWM, the bacteria were able to maintain similar growth to those found after a short incubation, whereas this capacity was significantly decreased in the reference medium (BHI) (*p* < 0.001).

### Coculture of *Pseudomonas aeruginosa* and *Staphylococcus aureus* in the *in vitro* Chronic Wound Medium

**FIGURE 2:**
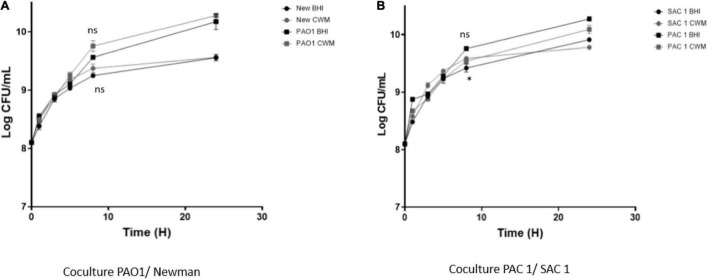
Growth curves of 1:1 coreference clinical **(A)** and clinical **(B)** strains of *S. aureus* and *P. aeruginosa* in CMW and BHI media. Cultures were sampled at the indicated time points, and the numbers of bacteria were estimated by CFU enumeration of *P. aeruginosa* and *S. aureus* on selective medium. Experiments were performed in three biological replicates; data points represent the average of these replicates, and error bars represent the standard deviation. Comparisons were performed with *t*-test. Significance was set to a *p* < 5%. ns, not significant; *p < 0.1.

Because of the frequent coisolation of *S. aureus* and *P. aeruginosa* in chronic wounds (Fazli et al., 2009), we compared the growth of the cocultured reference and clinical strains on BHI and CWM. *P. aeruginosa* PAO1*–S. aureus* Newman were able to grow in coculture in both media (Figure 2A). Moreover, no difference in bacterial growth was seen when the strains were cocultured (*p* = 0.32, NS). Concerning the clinical strains, both species were able to grow in a similar way in the two media (*p* = 0.21, NS) (Figure 2B). However, a slight decrease in SAC1 growth was observed in CWM (*p* = 0.05 at 8 h). Interestingly, the delay of the early exponential phase of SAC1, observed between 0 and 4 h when SAC1 was cultivated alone, was not present in coculture. Moreover, SAC1 growth was modified in CWM. Indeed, when cocultured with *P. aeruginosa* PAC1, SAC1 seemed to reach its growth plateau faster at 6 vs. 8 h) and with higher bacterial load (log 9 vs. log 8.7 in monoculture) compared with the growth of SAC1 alone (NS) (Figures 1C, 2B).

As observed with bacteria cultivated alone, the coculture in CWM significantly increased the morphotypes of SCVs and decreased the β-hemolysis of *S. aureus* and the presence of pigmentation of *P. aeruginosa* (*p* < 0.01) (Supplementary Table 3).

### Impact of Chronic Wound Medium on Early Biofilm Formation

To determine the impact of CWM on the bacterial virulence and particularly on biofilm formation, we used the BioFilm Ring Test^®^ on *P. aeruginosa* or *S. aureus* cultivated alone. First, we validated the performance of the BioFilm Ring Test^®^ using the CWM. At 1, 3, and 5 h, the control conditions showed that the microbeads were perfectly mobile in CWM, similar to results obtained in BHI medium (Supplementary Table 4). This result confirmed that the CMW (without human keratinocyte debris) could be used in this test.

**FIGURE 3:**
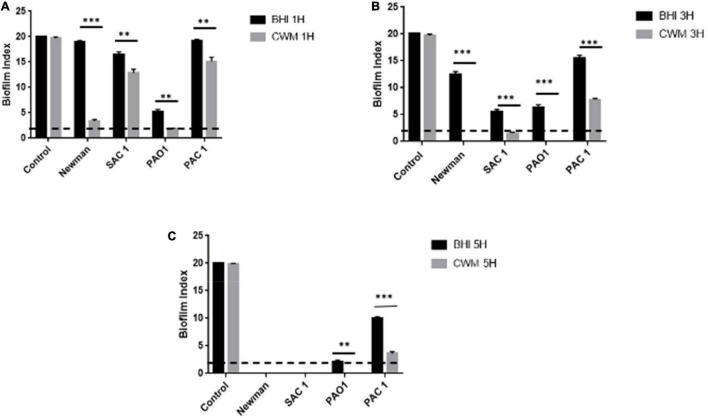
Kinetics of biofilm formation assessed by BioFilm Ring Test^®^ at 1 h **(A)**, 3 h **(B)**, and 5 h **(C)** for clinical and reference *S. aureus* and *P. aeruginosa* strains. Comparisons were performed with *t*-test. Error bars represent standard deviation. Significance was set to a *p* < 5%. The dotted horizontal line < 2 represents the cutoff for fixed biofilm. Control is well without bacteria. **p < 0.01; ***p < 0.001.

Second, we evaluated the kinetics of the early biofilm formation of both reference strains. After 1 h incubation, we observed that *S. aureus* Newman and *P. aeruginosa* PAO1 stuck very quickly to form a biofilm in CWM compared with BHI (BFI = 3.7 ± 0.2 vs. 19 ± 0.1 and 2 ± 0.2 vs. 6.7 ± 0.3, for *S. aureus* and *P. aeruginosa*, respectively) (*p* < 0.01) (Figure 3A). This significant difference was always observed at 3 h (*p* < 0.001) (Figure 3B). At 5 h, no difference was seen, showing that both reference strains have strongly adhered.

Finally, the study of the *S. aureus* clinical strain showed that SAC1 formed a biofilm in 5 h in the BHI medium (Figure 3C). This biofilm was constituted significantly faster in CWM than in BHI with a biofilm formed in 3 h (Figure 3B). The kinetics of biofilm formation was significantly different between the two media (Figures 3A,B) (*p* < 0.01), suggesting the influence of CWM on the biofilm induction. The same trend could be noted for *P. aeruginosa* PAC1. This strain developed a biofilm much later, and the biofilm remained incomplete even after 5 h in BHI (Figure 3C). However, at each measured point, the difference of BFI was significant (*p* < 0.01) with, for example, a BFI value of 12 ± 0.4 in BHI and 4.8 ± 0.3 in CWM at 5 h (*p* < 0.001).

### Mature Biofilm Biomass Is Enhanced in the Chronic Wound Medium

**FIGURE 4:**
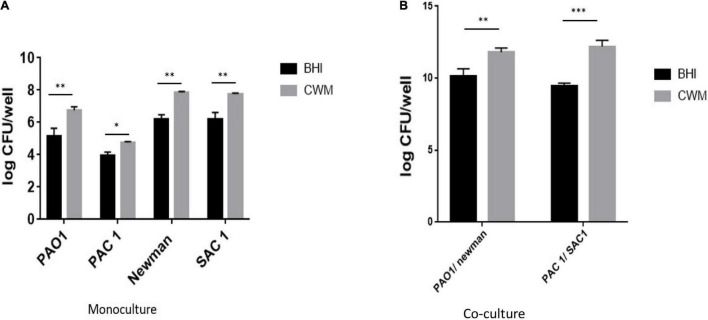
Quantification of biofilm of *S. aureus* and *P. aeruginosa* clinical and reference strains grown in monoculture **(A)** or 1:1 coculture **(B)**. Samples were tested in triplicate in two independent experiments. Comparisons were performed with *t*-test. Significance was set to a *p* < 5%. ***p < 0.001; **p < 0.01, *p < 0.1.

To corroborate the effect of the CWM on the early biofilm formation, we evaluated the mature biofilm living biomass of the different strains by quantifying the living bacteria present inside preformed biofilm. In monoculture, all strains presented a significantly higher number of living bacteria in the biofilm constituted in CWM than in BHI (*p* < 0.01) (Figure 4). For three of these isolates (Newman, SAC1 and PAO1), this augmentation was particularly significant, with a difference, on average, of a factor 10 (one log) (Figure 4A) (*p* < 0.01). The difference in number of live bacteria counted inside the preformed biofilm cultivated in CWM conditions for the PAC1 strain was more important compared with the same strain cultivated in the control medium but without significance (*p* = 0.074).

In coculture, the same trend was observed, with a significant increase in the number of living bacteria counted in preformed biofilm constituted in CWM than in BHI (*p* < 0.01) (Figure 4B). Interestingly, the combination of *S. aureus* and *P. aeruginosa* in CWM significantly increased the biofilm formation of *S. aureus* at the expense of *P. aeruginosa* (*p* < 0.01) (Supplementary Table 5).

Those results are correlated with the early biofilm measurements and corroborated that the CWM induced a faster and heavier biofilm.

### Bacteria in Chronic Wound Medium Are Less Virulent in a Nematode Killing Assay

**FIGURE 5:**
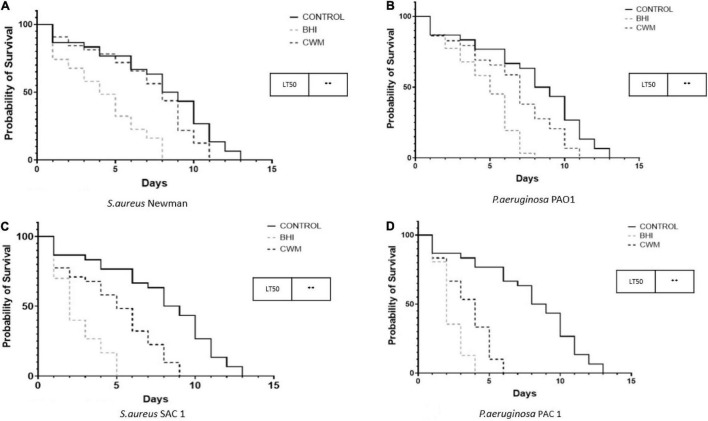
Nematode killing assays of monoinfections with reference **(A,B)** and clinical **(C,D)** strains of *S. aureus* and *P. aeruginosa* cultured in the different growing media.

Finally, we evaluated the impact of the CWM on bacterial virulence using a nematode killing model validated to study the host–pathogen interactions (Aballay and Ausubel, 2002; Messad et al., 2015; Pouget et al., 2021). In the *Caenorhabditis elegans* model, both *S. aureus* strains killed the nematodes more rapidly than the avirulent *E. coli* OP50 strain, irrespective of the culture medium used (*p* < 0.001) (Figure 5). However, a significant difference of LT50s was noted between Newman and SAC1 in BHI, with an increased nematode lifespan in the presence of Newman (LT50 = 6 ± 0.5 days vs. 5 ± 0.5 days, respectively; *p* < 0.001). When the *S. aureus* strains were cultivated in CWM, their virulence in the nematode models was significantly decreased compared with strains cultivated in BHI (6–8 ± 0.5 vs. 5–6 ± 0.5, respectively, *p* < 0.001; Figures 5A,C).

We observed the same trend for *P. aeruginosa* with the following: (i) PAO1 and PAC1 grown in BHI or CWM killed the nematodes more rapidly than the avirulent *E. coli* OP50 strain (*p* < 0.001); (ii) the LT50s were shorter for bacteria cultivated in BHI compared with those cultivated in CWM (2–3 ± 0.5 days vs. 4–5 ± 0.5 days, respectively; Figures 5B,D) (*p* < 0.001).

## Discussion

Microorganism behavior is highly dependent on the environment in which they evolve. Classically, in *in vitro* experiments, bacteria are grown in conventional culture media, a very artificial condition compared with the environment encountered by the bacteria in clinical situations. This condition influences the bacterial virulence, but this effect is currently far from the clinical conditions. The development of new *in vitro* models mimicking *in vivo* conditions remains an important challenge to improve our knowledge on the pathophysiology of the infections and to evaluate new therapeutic solutions.

In the chronic wound context, the environment encountered by microorganisms is very different from the general composition of classical microbiological culture media. Interfering factors specific to this clinical situation include the high bacterial diversity, the numerous bacterial interactions, the influence of the patient’s immune status with the modifications of the local pH, temperature, and tissue hypoxemia (inducing oxygen deprivation) (Dowd et al., 2008; Gardner et al., 2013; Smith et al., 2016; Rahim et al., 2017; Patel et al., 2019). All these elements modulate bacterial virulence and lead the microorganisms to form a complex polymicrobial biofilm community (Dowd et al., 2008). Previously, Sun et al. (2008) developed a chronic wound–like environment (the Lubbock model) adapted to both the growth of bacteria isolated from wounds and biofilm formation, aimed to mimic the microbiological environment of chronic wounds. However, this model did not closely reflect the human wound environment because of the use of animal products such as blood and plasma in concentrations that seemed not to be relevant with clinical data obtained in human wounds. Recently, we modified this medium by adding 10% glucose to mimic a DFU environment (Pouget et al., 2021) and observed a clear effect on *S. aureus* virulence, demonstrating the value of developing a more physiological medium. However, we believed that further improvements were necessary. We compared the composition of the medium described by Sun et al. and the clinical observations of chronic wounds. These ulcers are rarely irrigated due, in part, to vascular complication (Pendsey, 2010; Armstrong, 2011). A lower concentration of serum, preferred to plasma (Bandyopadhyay et al., 2006), and blood in the medium described above were more relevant; thus, we used 0.5% of hemolyzed human blood and 20% of heat-inactivated human serum to be as close as possible to the chronic wound environment. The use of Bolton broth was maintained with a concentration of 79.5%. Bolton broth is a peptone-based medium of animal origin and makes it possible to model the nutrients likely to be present in the early stages following debridement (e.g., damaged and degraded tissue and abundance of extracellular matrix compounds). The Bolton broth, serum, and blood thus form the three major constituents found in the wound bed, namely, red blood cells, serum, and damaged tissues. The Sun et al. model used a more acidic pH than those found in chronic wounds (7.2 vs. 8.5) (Gethin, 2007; Sun et al., 2008; Jones et al., 2015; Kumar and Honnegowda, 2015). Indeed, the physiological pH of the skin is acidic (pH 4–5) (Lambers et al., 2006). In the wound context, the pH significantly increases to reach approximately 8–9 (Gethin, 2007; Jones et al., 2015; Kumar and Honnegowda, 2015). This basic pH is one of the main characteristics of a non-healing wound. Usually, the healing process leads, by the local oxygen and nutrient requirements, to an acidification (Schneider et al., 2007; Svensson and Wahlström, 2017). The pH then returns to normal upon complete healing. However, in the case of chronic wounds, as there is no healing process, the pH remains approximately 8–9. We consequently buffered our medium at pH 8.0 with 10 mM HEPES/NaOH. Finally, to mimic the cellular and inflammatory environment present in the wound (Boukamp et al., 1988), we also added 1.10^6^/mL of human keratinocyte debris. Therefore, we proposed a new *in vitro* model more similar to a chronic wound environment.

To validate this model, we studied the interaction between *S. aureus* and *P. aeruginosa*, two of the main microorganisms present in chronic wounds (Serra et al., 2015). Coinfection by these two strains has been frequently described (Fazli et al., 2009) and shown to cause more severe infections than monoinfection (Hendricks et al., 2001; Pastar et al., 2013). However, difficulties in growing them together *in vitro* have delayed the understanding of their interaction. Indeed, it has been proven that in classical culture media, *P. aeruginosa* and *S. aureus* growth is not possible (Filkins et al., 2015). In this artificial environment, bacteria are mainly in competition for nutrients such as iron, and one is taking the advantage on the other one. In our study, the CWM gave reproducible and reliable results. *P. aeruginosa* and *S. aureus* were able to grow alone or cocultivated. Growth was different in CWM than in traditional laboratory growth medium (BHI). In monoculture, the species reached maximum cell density higher in BHI than in the CWM (*p* = 0.00103). More importantly, in coculture, contrarily to what was previously described (Palmer et al., 2005, 2007), *S. aureus* was not quickly eradicated by *P. aeruginosa* in either BHI or CWM: not only both species were able to coexist in both media, but also the coculture in CWM allowed *S. aureus* to reach a higher cell density. In particular, there was no early lag phase in the growth curve in coculture as observed in monoculture in CWM (Figure 2). Recent studies have reported that *P. aeruginosa* could have a protective effect for *S. aureus* (Price et al., 2020; Schurr, 2020). We confirm this trend, as this initial lag phase did not exist in coculture. In addition, the plateau reached after 24 h of coculture corresponds to a higher cell density (*p* = 0.00261). This indicates that the CWM influences the behavior of one or both of the bacteria species. As it concerns the growth ability of bacteria in chronic wound conditions, studies (Watters et al., 2015; Morgan et al., 2019) noted the same tendency on *P. aeruginosa* isolated from human wounds. One explanation of this current observation is that some key regulators of bacterial fitness could be inactivated. Chronic wound environment is also known to be stressful; some studies have investigated how it could impact bacteria particularly on certain metabolic pathways (Wei et al., 2019; Wang et al., 2020).

As the environment could represent a major source of stress for bacteria, we evaluated the role of CWM on biofilm formation and virulence. First, we examined the early adhesion, the first step of biofilm formation, and the mature biofilm formation by CFU counts of biofilm living biomass from monoculture and cocultures. The results obtained showed that adhesion was facilitated in the CWM compared with the BHI (Figure 3). Indeed, all the strains adhered more quickly in the CWM. This observation is important because the wound bed contains organic or inorganic nutrients on which bacteria get attached (Dunne, 2002). Those nutrients are basically not well represented in usual culture media. In the CWM, tissue lesions are mimicked by the serum, blood, and keratinocyte debris. Moreover, primary adhesion between bacteria to living or devitalized tissue is accomplished through specific molecular (lectin, ligand, or adhesin) docking mechanisms (Do et al., 2019). We could observe that the environment recreated by the CWM (supported by human proteins found in serum) can better relate to the initial bacterial adhesion steps that occur in clinical situations.

We also verified whether, beyond the early adhesion step, the CWM facilitates biofilm formation by measuring the living biomass present inside the biofilm after 24 h of incubation. The results corroborated the early adhesion evaluation confirming that CWM promotes the formation of biofilm (Figure 4). Indeed, the measurement of the total living bacteria present inside a preformed biofilm highlighted that in chronic wound environment, these bacteria were significantly found after 24 h of incubation. Globally, this indicates that the CWM medium is, first, compatible with biofilm studies. Second, we suggest that this environment induces a rapid, strong, and dense biofilm. Interestingly, these results are consistent with the behavior of the strains described in chronic wounds (Dowd et al., 2008; Smith et al., 2016), suggesting that this new medium is close enough to the clinical situation and could mimic the environment encountered by bacteria in these ulcers. Finally, we investigated the effect of the CWM on bacterial virulence. All *S. aureus* and *P. aeruginosa* strains tested were significantly less virulent in the CWM, compared with BHI (Figure 5). Thus, CWM is suitable for virulence tests, supporting our earlier studies showing that the environment present at the chronic wound level influences the strains’ virulence (Pouget et al., 2021). These observations are not the first highlighting the influence of the environment in which bacteria evolved on their virulence. It has been demonstrated that certain pathogens such as *Cutibacterium acnes* or even *Streptococcus pyogenes* can have a different virulence in a microenvironment similar to their ecological niche (Ngba Essebe et al., 2017; Borrel, 2019). With regard to *S. aureus* and *P. aeruginosa*, a chronic wound environment has been previously shown to alter the synthesis of virulence factors such as protein of the type IV secretion system (Watters et al., 2015) or that bacterial cooperation between several species could attenuate the virulence of these two major pathogens isolated in chronic wounds (DeLeon et al., 2014; Xiang et al., 2019). Thus, in this wound environment that mimics non-rich condition, bacteria seem to growth under stress, form a rapid and dense biofilm, and decrease their virulence, hijacking the immune defense system and maintaining the chronicity of the wound. Further studies must be done to evaluate the bacterial cooperation and regulation of biofilm on planktonic and sessile status.

This medium presents multiple benefits, notably in providing a means to study bacterial virulence and cooperation in a chronic wound environment. It also gives the possibility to modify different parameters. For example, to mimic DFU, we could vary the glucose concentration, as glucose is an important factor influencing wound healing (Roberts et al., 2015; Almaramhy et al., 2018) and bacterial behavior (Pouget et al., 2021). Moreover, as antibiotics are classically overused in this clinical situation, we could add different antibiotic concentrations and evaluate their impact on biofilm formation and bacterial virulence. Some authors have already highlighted and criticized the fact that the scientific community does not have a relatively standardized model to reliably study biofilm under *in vivo* conditions (Attinger and Wolcott, 2012; Coenye et al., 2018). Thus, this new medium contributes to this standardization to provide a more reliable model for research on chronic wounds and could help to lead to the discovery of future therapeutic molecules with antibiofilm strategy.

## Conclusion

In conclusion, we improved the *in vitro* wound-like model described by Sun et al. (2008) to try to better reflect the chronic wound environment. This study showed that the CWM, an affordable (approximately 100 euros/liter), reproducible, and reliable medium, allows the study of multiple bacteria species known to be present in chronic wounds and requiring in-depth study. In this model, we highlighted that the CMW had a strong impact on bacteria behavior, notably on their growth, interaction, biofilm formation, and virulence. Moreover, *S. aureus* and *P. aeruginosa* displayed synergism with an improved growth of *S. aureus* in the presence of *P. aeruginosa*. Taken together, these data suggest that virulent bacteria growing together in wounds have a main common objective to hijack the host immune defense by coaggregating symbiotically in a pathogenic biofilm (Duthie and Lorenz, 1952; Holloway et al., 1979) that participates considerably in the chronicity of the wound.

## Data Availability Statement

The original contributions presented in the study are included in the article/Supplementary Material, further inquiries can be directed to the corresponding author/s.

## Author Contributions

CPo, J-PL, and AS drafted the manuscript. All authors contributed to manuscript revision, read and approved the submitted version.

## Conflict of Interest

The authors were co-inventors of the CWM (European patent application EP21305337, filed on 18 March 2021). CPo was the recipient of a grant from Biofilm Pharma (Bourse CIFRE). CPo, TB, CPr, and JT were employed by Biofilm Pharma SAS.

## Publisher’s Note

All claims expressed in this article are solely those of the authors and do not necessarily represent those of their affiliated organizations, or those of the publisher, the editors and the reviewers. Any product that may be evaluated in this article, or claim that may be made by its manufacturer, is not guaranteed or endorsed by the publisher.
