# Supplementary material for: A Relevant Wound-Like in vitro Media to Study Bacterial Cooperation and Biofilm in Chronic Wounds
Source: Front Microbiol. 2022 Apr 6;13:705479. doi: 10.3389/fmicb.2022.705479 (PMC9019750; doi:10.3389/fmicb.2022.705479)
Supplement: Supplementary file 1 [file Data_Sheet_1.docx]

Supplementary Material

# Supplementary Figures and Tables

**Table S1**. Phenotypical modifications of *S. aureus* and *P. aeruginosa* cultivated in BHI and CWM for 72-hrs.

|  | **Culture in BHI Medium** | | | | | **Culture in CWM** | | | | **p** (**BHI vs CMW)** | | | | |
| --- | --- | --- | --- | --- | --- | --- | --- | --- | --- | --- | --- | --- | --- | --- |
| **Strains** | Newman | SAC1 | | PAO1 | PAC1 | Newman | SAC1 | PAO1 | PAC1 | Newman | SAC1 | PAO1 | PAC1 |  |
| **%SCV*** | 12 (±0.8) | 6 (±0.5) | 5 (±0.6) | | 4 (±0.8) | 22 (±0.9) | 21 (±0.4) | 16 (±0.5) | 13 (±0.6) | 0.01 | 0.006 | 0.009 | 0.025 |  |
| **%Pigment** | - | - | 99 (±0.8) | | 96 (±0.7) | - | - | 68 (±0.7) | 72 (±0.6) | - | - | 0.0009 | 0.001 |  |
| **% β hemolysis** | 88 (±0.7) | 79 (±0.8) | - | | - | 54 (±0.3) | 51 (±0.4) | - | - | 0.0003 | 0.0006 | - | - |  |

*SCV, small colony variants

**Table S2**. Comparison of maximal density obtain after growth of *S. aureus* and *P. aeruginosa* cultivated in BHI and CWM for 24-hrs versus 6 weeks.

|  | **Culture in BHI Medium** | | | | **Culture in CWM** | | | | **p** (**24-hrs vs 6 weeks)** | | | |
| --- | --- | --- | --- | --- | --- | --- | --- | --- | --- | --- | --- | --- |
| **Strains** | Newman | | PAO1 | | Newman | | PAO1 | | BHI | | CWM | |
| **Incubation Time** | 24-hrs | 6 weeks | 24-hrs | 6 weeks | 24-hrs | 6 weeks | 24-hrs | 6 weeks | Newman | PAO1 | Newman | PAO1 |
| **Log max density** | 9.2 (±0.3) | 6 (±0.5) | 9.5 (±0.4) | 5.2 (±0.8) | 8.5 (±0.2) | 8.3 (±0.3) | 9.3 (±0.4) | 9.4 (±0.3) | 0.001 | 0.0009 | NS | NS |

*NS, not significant

**Table S3**. Phenotypical modifications of *S. aureus* and *P. aeruginosa* co-cultivated in BHI and CWM for 72-hrs.

|  | **Culture in BHI Medium** | | | | **Culture in CWM** | | | | **p** (**BHI vs CMW)** | | | |
| --- | --- | --- | --- | --- | --- | --- | --- | --- | --- | --- | --- | --- |
| **Coculture** | Newman/PAO1 | | SAC1/PAC1 | | Newman/PAO1 | | SAC1/PAC1 | |  |  |  |  |
| **Strains** | Newman | PAO1 | SAC1 | PAC1 | Newman | PAO1 | SAC1 | PAC1 | Newman | PAO1 | SAC1 | PAC1 |
| **%SCV*** | 10 (±0.2) | 5 (±0.4) | 6 (±0.2) | 4 (±0.4) | 68 (±0.7) | 52 (±0.6) | 72 (±0.8) | 54 (±0.5) | 0.0009 | 0.0019 | 0.0003 | 0.0014 |
| **%Pigment** | - | 90 (±0.6) | - | 89 (±0.8) | - | 66 (±0.8) | - | 63 (±0.9) | - | 0.0011 | - | 0.0008 |
| **% β hemolysis** | 86 (±0.4) | - | 78 (±0.5) | - | 47 (±0.7) | - | 42 (±0.6) | - | 0.00045 | - | 0.0004 | - |

*SCV, small colony variants

**Table S4**. Early kinetics biofilm formation in BHI (control condition) and CWM at 1-hr (A), 3-hrs (B) and 5-hrs (C). The presence of the ring in controls in CWM showed that CWM is compatible with the Biofilm Ring Test without affecting the mobility of the beads.

**Table S5.** Proportion of *S. aureus* and *P. aeruginosa* in polymicrobial biofilm after co-culture in BHI and CWM.

|  | **Culture in BHI Medium** | | | | **Culture in CWM** | | | | **p** (**BHI vs CMW)** | | | |  |
| --- | --- | --- | --- | --- | --- | --- | --- | --- | --- | --- | --- | --- | --- |
| **Coculture** | Newman/PAO1 | | SAC1/PAC1 | | Newman/PAO1 | | SAC1/PAC1 | |  |  |  |  | |
| **Strains** | Newman | PAO1 | SAC1 | PAC1 | Newman | PAO1 | SAC1 | PAC1 | Newman/PAO1 | | SAC1/PAC1 | |  |
| **% Biofilm** | 45 (±0.2) | 55 (±0.3) | 40 (±0.5) | 60 (±0.4) | 60 (±0.4) | 40 (±0.2) | 68 (±0.3) | 32 (±0.5) | 0.003 | | 0.00075 | |  |
